# The Cryo-EM structure of the CorA channel from *Methanocaldococcus jannaschii* in low magnesium conditions

**DOI:** 10.1016/j.bbamem.2015.06.002

**Published:** 2015-10

**Authors:** Robert M. Cleverley, James Kean, Chitra A. Shintre, Clair Baldock, Jeremy P. Derrick, Robert C. Ford, Stephen M. Prince

**Affiliations:** Faculty of Life Sciences, University of Manchester, Oxford Road, Manchester M13 9PL, UK

**Keywords:** TmCorA, *Thermotoga maritima* CorA, MjCorA, *Methanocaldococcus jannaschii*, ATP, adenosine tri-phosphate, TM, trans-membrane, RMS, root mean square, DDM, dodecylmaltoside, SAXS, Small Angle X-ray Scattering, Cryo-EM, Cryo-Electron Microscopy, FSC, Fourier Shell Correlation, C_α_, amino acid α carbon, S, scattering vector, q, momentum transfer, ω,ϕ,κ, polar rotation angles, α,β,γ, Eulerian rotation angles, u,v,w, Patterson vector coordinates, Ion channel, Channel gating, Cryo-electron microscopy, Membrane protein

## Abstract

CorA channels are responsible for the uptake of essential magnesium ions by bacteria. X-ray crystal structures have been resolved for two full-length CorA channels, each in a non-conducting state with magnesium ions bound to the protein: These structures reveal a homo-pentameric quaternary structure with approximate 5-fold rotational symmetry about a central pore axis. We report the structure of the detergent solubilized *Methanocaldococcus jannaschii* CorA channel determined by Cryo-Electron Microscopy and Single Particle Averaging, supported by Small Angle X-ray Scattering and X-ray crystallography. This structure also shows a pentameric channel but with a highly asymmetric domain structure. The asymmetry of the domains includes differential separations between the trans-membrane segments, which reflects mechanical coupling of the cytoplasmic domain to the trans-membrane domain. This structure therefore reveals an important aspect of the gating mechanism of CorA channels by providing an indication of how the absence of magnesium ions leads to major structural changes.

## Highlights

• CorA channels regulate magnesium ion concentration in the bacterial cytoplasm.
• We report the first structural data on CorA when depleted of magnesium ions.
• The absence of magnesium ions leads to conformational changes in the channel.
• CorA structure is influenced by ionic bonds between channel domains.
• CorA structure is influenced by monovalent ions binding to cytoplasmic domains.

## Graphical abstract

**Figure:**
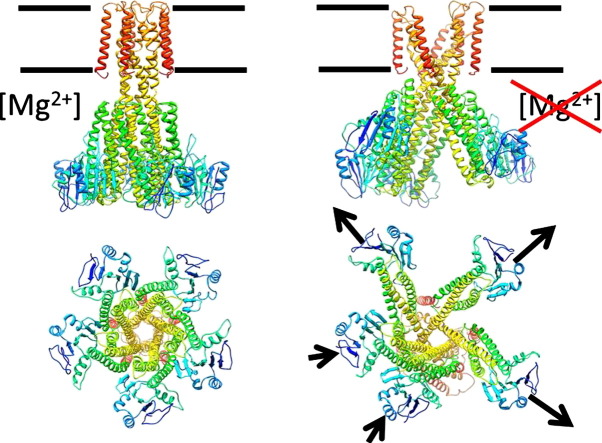

## 1 Introduction

Magnesium is the most abundant divalent cation in the cytoplasm of living cells [1]. Mg^2+^ forms complexes with nucleic acids and proteins, is an important co-factor in many enzyme catalysed processes and the hydrolysis of Adenosine Tri-Phosphate (ATP) requires Mg^2+^ as a co-factor [1]. In *Escherichia coli* (*E. coli*) the free cytoplasmic concentration of Mg^2+^ has been estimated at 2 mM [2]; the affinity of ATP for Mg^2+^ has been measured as 100 μM [3], therefore 95% of the ATP in the cell is found in complex with Mg^2+^. This implies that Mg^2+^ concentration is an important regulatory factor in the bacterial cell. The CorA proteins are ion channels having a major role in the flux of magnesium ions across the cytoplasmic membrane in prokaryotes [4]. Their physiological importance was underscored by their identification as virulence factors in the pathogens *Leishmania major* [5] and *Salmonella typhimurium* [6]. A CorA homologue, Mrs2, is present in the mitochondrial membranes of eukaryotes [7] and bacterial CorA can functionally compensate for a Mrs2 mutant in yeast [8]. Despite low sequence identity (< 25%) the yeast Mrs2 structure is similar to that of bacterial CorA [9].

**Fig. 1:**
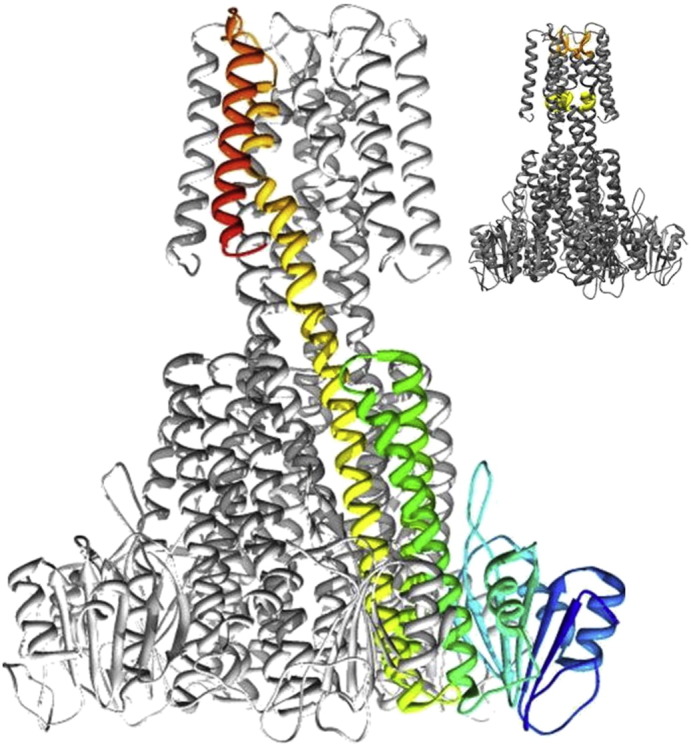
The crystal structure of MjCorA: The crystal structure of MjCorA [12] rendered with UCSF Chimera [34] (PDB ID: 4EV6). The protein is shown in a cartoon representation with β-sheets and α-helices highlighted as rounded ribbons. One of the five protomer subunits is picked out in colour. The colouring scheme indicates the sequence of the peptide from amino-terminus (dark blue) through cyan, green yellow and orange to red at the carboxy-terminus. With reference to the text; (1) The N-terminal domain is therefore coloured in shades of blue/cyan, (2) the conical domain in green/yellow and (3) the TM domain in orange/red. Inset: A similar image of the crystal structure with the location of YGMNF motifs picked out in orange and TMVTT motifs picked out in yellow.

The crystal structures of the CorA channels from *Thermotoga maritima* (TmCorA) [10], [11] and *Methanocaldococcus jannaschii* (MjCorA) [12] have been determined. CorA channels are homo-pentameric with a large cytoplasmic domain and a smaller trans-membrane (TM) domain. The structure of each monomer of the MjCorA channel (Fig. 1) consists of (1) an N-terminal domain containing both α-helical and β-sheet secondary structures; (2) a conical domain constructed from a triple α-helical bundle linked by two short turns. The last, pore lining, helix of this bundle is continuous into (3) a helix-turn-helix motif embedded in the membrane. The carboxy-terminal helix forms the lipid-facing interface and both the C-terminus and the N-terminus of the CorA monomer are located in the cytoplasm.

For TmCorA and MjCorA the initial strategy adopted in the solution of the native crystal structure involved crystallization of the full length channel from solution conditions consisting of a high Mg^2+^ ion concentration and an alkaline pH. Alongside the structure of the full-length channel the structure of an N-terminal truncation comprising the cytoplasmic domain was also determined. For both TmCorA and MjCorA the fold of the cytoplasmic domain was the same in the full-length channel and the truncated structure, as demonstrated by the use of the cytoplasmic domain in phasing the crystal structure of the full-length channel by molecular replacement. However for both channels the cytoplasmic domains alone do not form the same quaternary structure as that of the full length channel, which indicates that the TM domain is a key determinant of the pentameric structure. Both MjCorA and TmCorA full-length crystal structures adopt near C_5_ molecular symmetry [11], [12].

The TmCorA and MjCorA channel structures are assumed to be in a closed, non-conducting state, as both structures were crystallized in the presence of excess magnesium. Gating of the CorA channel to modulate ion conduction is thought to occur through conformational changes in the CorA protein which in turn are moderated by the binding of divalent ions. The Mg^2+^ ion has a stable solvation shell [1], [13] and the selectivity of the CorA channel is thought to reside in the recognition of wholly or partially solvated Mg^2+^ ions by the membrane spanning domain [14]. The inset to Fig. 1 shows the location of YGMNF motifs in the first TM helix of MjCorA. A dehydrated Magnesium ion is assigned here in the MjCorA crystal structure coordinated with the peptide bond oxygen of Glycine 278 and the side chain amide oxygen of Asparagine 280. A Threonine rich motif (TMVTT) is also highlighted. A hydrated Mg^2+^ ion is assigned in this region in the crystal structure. The first coordination shell radius for solvated Mg^2+^, measured as the mean ion to water oxygen distance in a molecular dynamics study, is 2.2 Å [13]. The pore diameter shown in the crystal structure exceeds 4.4 Å between the aforementioned amino acid motifs. CorA channels have a gate formed by an annulus of hydrophobic residues coincident with the inner membrane surface and [15], [16], in the structures of CorA channels determined to date the dimensions of this gate region would block the passage of magnesium ions through the channel. CorA channel conduction is moderated by divalent cations. Mg^2+^ ion binding sites have been identified in the cleft between adjacent TmCorA channel monomers. A number of these ions are coordinated between aspartate, glutamate and histidine residues from both N-terminal domains and conical domains. This polydentate liganding of Mg^2+^ ions indicates that the CorA cytoplasmic domain is effectively a chelator of Mg^2+^. In the MjCorA crystal structure a larger number of Mg^2+^ sites are assigned but fewer are assigned to chelating sites. For MjCorA one face of the cleft between each pair of protomer domains in the cytoplasm has an “acidic surface” formed from a high density of Asp and Glu residues, a varying number of Mg^2+^ are assigned to be decorating each of these surfaces in the MjCorA crystal structure. These binding sites are distant from the central pore and are likely to regulate channel function in response to magnesium levels in the cytoplasm.

In an attempt to resolve regulatory features of CorA channels a mutant of TmCorA, where the N-terminus of the protein was truncated and two basic residues in the conical domain were exchanged to alanine has been crystallized in the presence of Mg^2+^ and, separately, in the presence of monovalent cations only [17]. Both of the crystal structures of the mutant TmCorA showed small changes from the earlier structure of wild type TmCorA in excess Mg^2+^. There are also minor differences between the crystal structures of magnesium-bound wild type and magnesium-free mutant CorA. A molecular dynamics study of wild type TmCorA conducted in the absence of Mg^2+^ ions indicates a large movement of the cytoplasmic domain relative to the trans-membrane domain [17], but currently there is no experimental structural data to support this.

In order to further analyse the open state of the CorA channel, we have integrated lower-resolution studies of MjCorA using Small Angle X-ray Scattering and Single Particle Cryo-Electron Microscopy in the absence of magnesium and combined these with low resolution X-ray crystallographic data from crystals grown in low pH conditions disfavouring magnesium binding.

## 2 Materials & methods

All chemicals were obtained from Sigma-Aldrich (Fancy Road, Poole, Dorset BH12 4QH, UK) unless stated. *E. coli* bacterial strains were obtained from Novagen/Merck Chemicals (Boulevard Industrial Park, Padge Road, Beeston, Nottingham, NG9 2JR, UK). All columns were purchased from GE Healthcare (The Grove Centre, White Lion Road, Amersham, Bucks, HP7 9LL, UK); chromatography was undertaken using a GE Healthcare AKTA Fast Protein Liquid Chromatography (FPLC) rig.

### 2.1 Protein purification

Detergent solubilized MjCorA was purified using Immobilized Metal Affinity Chromatography, tag cleavage with Chymotrypsin and Size Exclusion Chromatography as described earlier [18]. The final buffer after the size exclusion step was 200 mM NaCl, 20 mM Tris/HCl pH8, 0.04% dodecylmaltoside (DDM). Purified MjCorA samples were flash cooled using liquid nitrogen and stored at − 70 °C.

### 2.2 Crystal preparation

MjCorA crystals were grown by vapour diffusion by mixing 1 μL of purified MjCorA at 3.2 mg/ml with 1 μL of well solution consisting of 0.1 M sodium citrate pH 3.5, 50 mM magnesium sulphate, 50 mM cobalt chloride, 0.1 M sodium chloride, 13%(w/v) PEG 4000 and 0.9% (w/v) β-octylmaltoside. Crystals used for recording diffraction typically grew to dimensions of 0.2 × 0.4–1.0 × 0.1 mm^3^ with growth generally complete 1–2 weeks after nucleation. Crystals were cryo-protected by layering 5 μL of 0.1 M sodium citrate pH 3.5, 0.1 M magnesium sulphate, 0.1 M sodium chloride, 13% (w/v) PEG 4000, 15% (v/v) glycerol onto the hanging drops. After approximately 1 minute crystals were looped out and cryo-cooled by plunging into liquid nitrogen.

### 2.3 X-ray crystallography

**Fig. 2:**
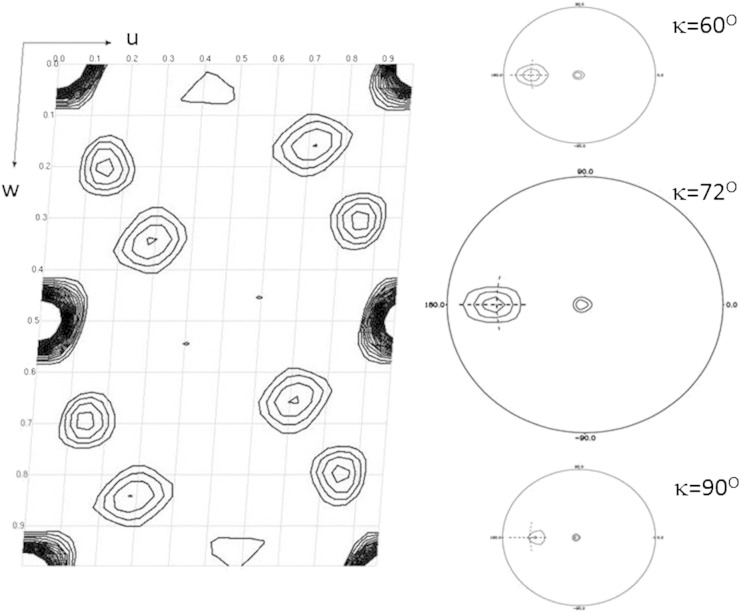
Analysis of MjCorA crystallized at low pH: Left, the v = 0 section of the native Patterson map calculated from the crystallographic data of MjCorA. Data to a resolution of 12 Å and of significance F > 2σ(F) were included in the calculation. The u Patterson axis runs horizontally and the w axis runs down the page, gridlines are at fractional coordinate intervals of 0.1. A peak of 0.71 × the origin peak height is found at u,v,w = 0,0,0.5. Right, the κ = 60;72;90° sections (top; middle; bottom, respectively) of the self-rotation function calculated from the crystallographic data of MjCorA in polar coordinates (0 < ω < 90° is plotted radially, − 180 < ϕ < 180° around the circumference). Data to a resolution of 12 Å were included in the calculation. The crosshairs intersect at ω = 67° ϕ = 180°. Calculation by the CCP4 program POLARRFN [31] with an integration radius of c/2 (= 82 Å).

Although relatively large the MjCorA crystals often showed multiple lattices or split diffraction profiles during screening. Crystallographic diffraction data were recorded from a single cryo-cooled crystal at the I24 (microfocus) beamline of the Diamond Light Source at a wavelength of 0.9778 Å with a 10 × 10 μm beam. Optimal locations for illumination were identified by taking test exposures using the grid crystal centring facility implemented at I24. The crystal was rotated by one degree for each data image, to ameliorate the effects of radiation damage the crystal was re-centred at a different grid location after each 30° segment of data. 160 images comprising two wedges of 130 and 30° were integrated over the resolution interval 63.2–9.0 Å. The diffraction images were indexed and integrated with MOSFLM [19] and the reflection intensities merged with SCALA [20], [21]. Spacegroup analysis was carried out with the program POINTLESS [21], [22] and determined to be P2_1_ with a unit cell of a = 116.2 Å, b = 151.0 Å, c = 163.2 Å, β = 94.25°. The crystallographic data were 94(96)% complete with an R_merge_ of 13.9(24.6)%, where figures in parentheses indicate the values for the outermost resolution shell (9.49–9.0 Å). The data were highly anisotropic with the highest observed diffraction spot resolution at approximately 7.5 Å. Analysis of the native Patterson function (Fig. 2) showed a large peak (u,v,w = 0.0,0.0,0.5; 71% of the origin peak height) indicating the presence of translational non-crystallographic symmetry similar to that reported for TmCorA crystals [23]. A number of attempts were made to use either the MjCorA crystal structure, the Cryo-EM maps reported herein, or combinations of these to phase the low resolution crystallographic data set. These attempts were unsuccessful in all cases, presumably because the low resolution of the crystallographic data set did not allow the position of any of the molecular replacement models to be determined with sufficient precision within the unit cell.

### 2.4 Small Angle X-ray Scattering (SAXS)

Data were collected at station I22 of the Diamond Light Source. A 200 μL sample of MjCorA at a single concentration of approximately 8 mg/ml in size exclusion buffer was injected between the mica windows of the sample cell. 200 1 s exposures were recorded. The flow through from the final centrifugal concentration step in the MjCorA preparation was used to record 200 1 s exposures both before and after the MjCorA sample for later subtraction. The MjCorA SAXS profile extended over the momentum transfer (q) range of 1.094 × 10^− 2^ < q < 3.703 × 10^− 1^ Å^− 1^ corresponding to information in the resolution interval of 575.3 > S^− 1^ > 16.9 Å (S denotes the scattering vector). Individual scattering images were reduced to 1-dimensional I(q) profiles using beamline software. The 200 individual scattering profiles derived from each 1 s exposure of MjCorA in buffer were averaged in blocks of 10 and compared: This indicated no progressive increase in the scattering intensity at lower resolution as would be consistent with radiation induced protein aggregation. The SAXS profiles from all of the exposures were therefore averaged to give the final MjCorA in buffer SAXS profile. The averaged scattering profiles of buffer alone recorded either side of the protein sample were not significantly different. A buffer profile for subtraction was obtained by averaging the 200 profiles recorded after the MjCorA in buffer data set. Averaging of data profiles to give combined I(q) and error estimates, σI(q), was carried out using spreadsheet software, subtraction of the buffer profile from the MjCorA in buffer profile was done using PRIMUS [24]. An intensity file for *ab-initio* shape determination with the program DAMMIN [25] was produced using the program GNOM [26], *Ab-initio* modelling was carried out assuming 5-fold rotational point symmetry, a cylindrical starting model and with a prolate overall structure expected. 16 *ab-initio* models were generated for which the agreement of the calculated scattering profile with the measured I(q) was in the range of 0.60 < χ < 0.66. The most probable model of the set was determined by the program DAMSUP [27] (χ = 0.63) by comparison within all of the 16 models determined.

### 2.5 Cryo-Electron Microscopy (Cryo-EM)

**Fig. 3:**
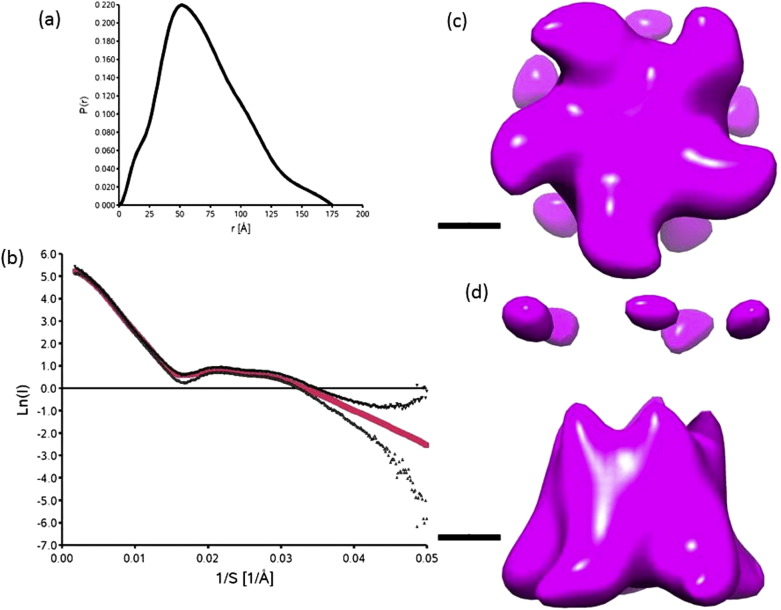
The SAXS model: (a) The pair distribution function of the SAXS data determined during data reduction with GNOM [26]. (b) The agreement of the most probable DAMMIN [25] model of MjCorA (χ = 0.63); raw data are plotted as the natural logarithm of I + σ(I)/I(▾) and I − σ(I)/I(▴) against the scattering vector 1/S (Å^− 1^), the model gives the calculated scattering curve shown in red. (c) Top (cytoplasmic) and side (d) views of an envelope representation of the DAMMIN model of MjCorA. Envelopes are rendered using the program UCSF Chimera [34] with the contour level calculated to enclose a volume corresponding to a mass of 272 kDa, accompanying scale bars are 25 Å long.

20 μL aliquots of purified MjCorA were thawed and spun in a micro-centrifuge. 3 μL of the supernatant was pipetted onto Quantifoil R 1.3/2 holey carbon-coated EM grids, blotted using Whatman No.1 filter paper (2 × 1 s blots) at 90% humidity and then cooled in liquid ethane using a Vitrobot plunge freezing system (FEI, Hillsboro, Oregon U.S.A.). Cryo-EM was performed using a Tecnai F20 200 kV EM operating in low dose mode at 200 kV. Micrographs were recorded using a Gatan 4k × 4k CCD with defocus values in the range 4.8–3.3 μm and an effective pixel size of 3.5 × 3.5 Å. An envelope for particle picking was generated from the single most probable DAMMIN model using the EMAN [28] subroutine *pdb2mrc* and a resolution of 32 Å (shown in Fig. 3c, d). 44,064 particles were picked from the hole regions of 28 micrographs using model based particle picking as implemented in the *boxer* routine of EMAN. The box size was 168 × 168 Å for both picking and refinement. Supplementary Fig. 1 shows a representative raw micrograph with boxes. In the initial stages of refinement the Contrast Transfer Function correction was carried out using the I(s) profile calculated from the DAMMIN model using CRYSOL [29]. The initial model was the envelope derived from SAXS and C_5_ symmetry was imposed during refinement. The crystallographic I(hkl) and σI(hkl) data were averaged into 150 resolution bins using the CCP4 program SFTOOLS and merged with the MjCorA SAXS data using PRIMUS [24] to generate a I(S) curve over the extended range of 575.3 > S^− 1^ > 9.0 Å for improved Contrast Transfer Function correction in the final stages of Cryo-EM refinement. To estimate the resolution of Cryo-EM maps the particle data was split into two halves and the final section (4 cycles) of refinement were repeated without masking or filtering. The Fourier Shell Correlation (FSC) was calculated for the resulting pair of models and fitted to a curve of the form FSC(S) = 1/(1 + exp(aS-b)) [30] and the resolution was calculated from b/a.

#### 2.5.1 Accession numbers

The final experimental Cryo-EM map has been deposited in the Electron Microscopy Data Bank with the accession code EMD-2626.

## 3 Results

### 3.1 Quaternary structure determination from crystallographic data

In order to assess the oligomeric state of MjCorA a self-rotation function was calculated using POLARRFN [31] (Fig. 2) in polar coordinates (ω,ϕ,κ), giving a peak close to the κ = (360/5)° section consistent with a C_5_ axis of symmetry. This peak was smeared between 60 < κ < 90° sections indicating correlations over multiple self-rotation values. The self-rotation function analysis of the crystallographic data supported a pentameric MjCorA and this quaternary structure was assumed for the succeeding Scattering and Microscopy analysis. The smearing of the peak in the self-rotation function also indicates that the C_5_ rotational symmetry of the channel is not exact.

### 3.2 Small Angle X-ray Scattering analysis and initial model determination

The structural information on the conformation of CorA in solution gained by solution SAXS is shown in Fig. 3. A Radius of gyration (R_g_) of 53.2 Å was calculated from the Guinier plot for the detergent solubilized MjCorA oligomer. The maximum diameter of the particle was estimated as 175 Å in the calculation of the pair distribution function (shown by the intercept at P(r) = 0 in Fig. 3a). Dummy atom modelling was conducted over the full q interval rather than limited to a resolution truncated to R_g_/8 where the relationship between Molecular Mass and the hydrated particle volume is approximately linear [32], and internal scattering variations within the particle can be neglected. The DAMMIN model was reduced to a resolution of 32 Å before use in particle picking or as a starting Cryo-EM envelope as described in Section 2.5. Agreement between the SAXS data and the dummy atom model is good over the recommended interval (0 < S < 2.4 × 10^− 2^ Å, Fig. 3b). The inclusion of data with a large signal/noise ratio at higher q may have led to systematically lower values of χ for the DAMMIN dummy atom modelling.

The agreement of the most probable *ab-initio* SAXS model is shown alongside the scattering data in Fig. 3b alongside the smoothed envelope representing the model itself (Fig. 3c, d). Other models in the DAMMIN set varied in the diameter of the large conical domain (Fig. 3c) and the overall height of the model (Fig. 3d).

### 3.3 Single Particle Cryo-Electron Microscopy

**Fig. 4:**
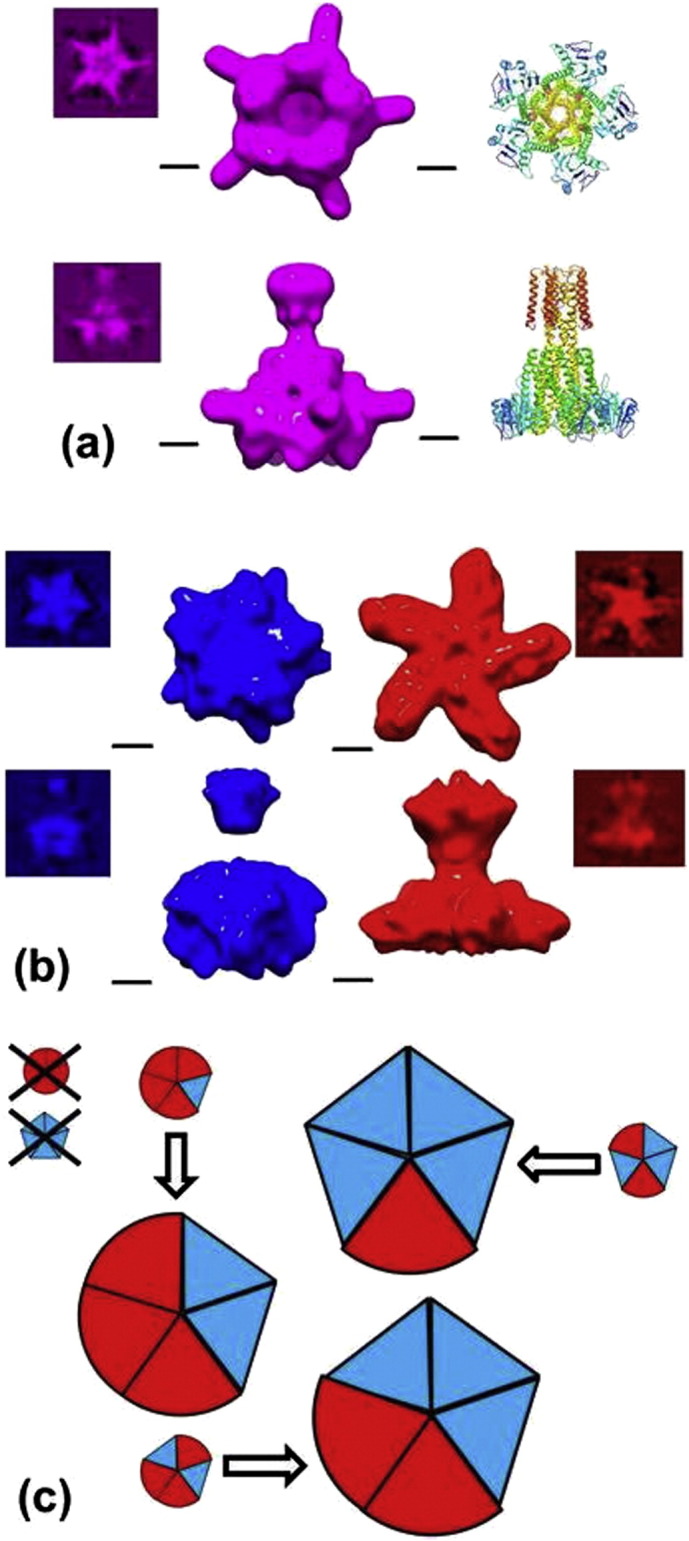
The evolution of the Cryo-EM model: (a) Top and side views of the refined model of MjCorA with C_5_ molecular symmetry imposed shown alongside the crystal structure of MjCorA (representation as in Fig. 1). (b) Top and side views of multi-refined C_5_ symmetric MjCorA models shown alongside one another. For (a) and (b) raw class averages are shown alongside the relevant map. (c) A schematic representation of the refinements of segmented combinations of the models shown in (b) which were explored. Envelopes are rendered using the program UCSF Chimera [34] with the contour level calculated to enclose a volume corresponding to a mass of 272 kDa, all scale bars are 25 Å long. In the schematic diagram the sizes of the individual models are illustrative of the number of particles assigned to each model in multi-refinement. The arrows indicate the convergence of the envelopes during multi-refinement. Taut model elements are indicated in blue, relaxed in red.

A C_5_ symmetric Cryo-EM envelope was refined from 44,064 particles which showed a narrow, cylindrical base fused to a widening conical top consistent with the gross structure expected of CorA (Fig. 4a). The refined model indicated a resolution of 16 Å at a FSC = 0.5 [33]. However the model also displayed features that could not be interpreted readily, namely narrow radial projections in the regions assigned to the cytoplasmic domain. The model did not allow the hand of the structure to be assigned which, given the resolution indicated by the FSC and the availability of a crystallographic model, should be achievable. Therefore the possibility of the presence of multiple structures in the single particle data was explored.

Two separate DAMMIN models were selected from the 16 refined versions each having different diameters of the cytoplasmic domain; envelopes generated from these were used as starting envelopes in a multi-refinement in EMAN. The multi-refinement implemented in EMAN proceeds in two stages (i) the allocation of particles to particular envelopes and (ii) refinement of each envelope against their respective pool of particles. Stage (i) is carried out until convergence is reached followed by stage (ii). For this multi-refinement process C_5_ symmetry was imposed and at convergence two clearly distinct envelopes resulted (Fig. 4b): 16,383 particles were assigned to a compact “taut” form with a low radius of both cytoplasmic and TM domains and 27,681 were assigned to the alternative model which showed a “relaxed” form with conical flared density distributions for both Cytoplasmic and TM domains. These maps were compared with the MjCorA crystal structure by docking a map calculated from the C_α_ coordinates of Protein Data Bank entry 4EV6 (PDB ID:4EV6) at a resolution of 25 Å using the program UCSF Chimera [34]. For the taut map the calculated correlation coefficient was 0.773(0.771) and for the relaxed map 0.803(0.801), where values in parentheses indicate the correlation coefficient of the Cryo-EM map with the map calculated from the MjCorA crystal structure after inversion of the hand of the map. Closer examination of the correspondence of the C_5_ multi-refined maps with the MjCorA structure indicated that neither map could accommodate the unmodified crystal structure: The N-terminal domain could be recognized in the relaxed map whilst the remaining conical and TM domains more closely resembled the taut map. As the chirality of these refined maps could be recognized by eye with reference features in the crystal structure these maps were judged to be an improvement on the initial single C_5_ map.

Fitting either of the C_5_ multi-refined maps would require a modification of the crystal structure. This implied that at least two conformations of the MjCorA protomer were present in the Cryo-EM data. Therefore the alternative possibility of asymmetric structure(s) was examined. The starting envelopes for these refinements were assembled by selecting segments from the taut-state and relaxed-state C_5_ multi-refined maps and combining segments to make an array of starting envelopes as follows ttttt; ttttr; tttrr; ttrtr; ttrrr; trtrr; trrrr & rrrrr where “t” denotes a segment extracted from the taut map and “r” from the relaxed map. As C_5_ molecular symmetry was broken by the majority of these envelopes, refinements were carried out at coarser angular sampling intervals (15° instead of 9°) to determine the most representative envelope(s).

The general strategy was again to use multi-refinement, to examine the numbers of particles assigned to each of the envelopes and to compare the similarity of the refined envelopes. The similarity was compared by summing the area under pairwise FSC correlation curves, larger values indicating more similar refined envelopes. In stage (i) of the multi-refinement process it was clear that when both asymmetric (C_1_) and symmetric (C_5_) envelopes were present in the multi-refinement very few particles (< 10%) were assigned to the symmetric envelopes: the ttttt and rrrrr envelopes were therefore discounted at an early stage. Also at stage (i) within the asymmetric envelopes those of the type ttrtr and trtrr where isolated t domain types were present in the starting envelopes, were disfavoured in terms of assigned particle numbers. The stage (i) and (ii) refinements of the remaining starting envelopes, all converged to structures of the form ttttr, tttrr or ttrrr. These exploratory refinements are illustrated schematically in Fig. 4c.

### 3.4 Calculation of the experimental Cryo-EM envelope

**Fig. 5:**
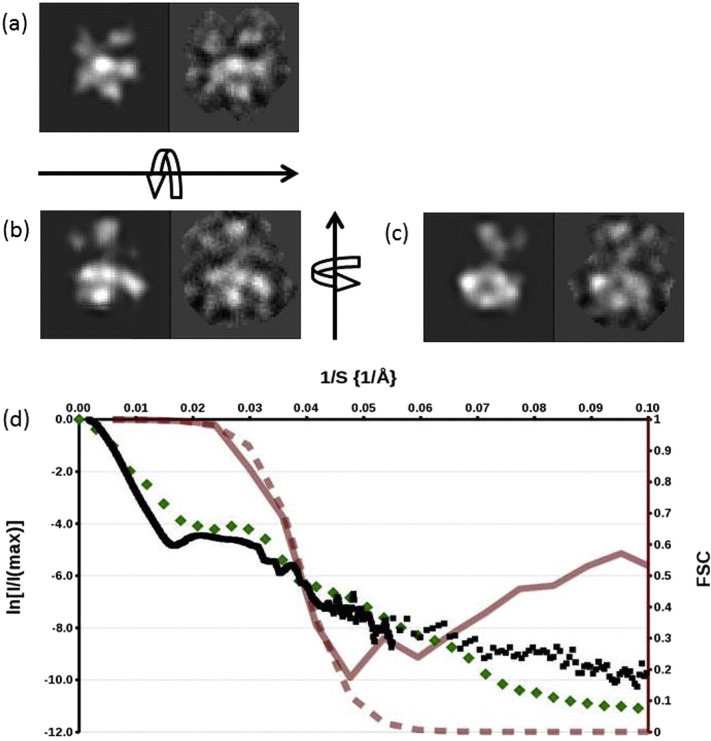
The consistency of the experimental Cryo-EM model. (a–c) Paired back projections (left) and masked class averages (right) from the final round of refinement. Orthogonal views are shown with a view down the channel axis topmost. (d) A composite plot showing the agreement of the final experimental Cryo-EM map with the scattering and crystallographic data, and the resolution and of the map. SAXS data merged with spherically averaged crystallographic data are shown as black squares mapping to the left hand axis: These data are plotted as the natural logarithm of the normalized intensity. The sharpened structure factor from the final map (after application of a Debye–Waller sharpening “B” factor of magnitude 361) is plotted with green diamonds. The semi-transparent solid, red line maps to the right hand ordinate axis. The line connects the Fourier Shell Correlation (FSC) points of the unmasked, unfiltered, final map plotted against 1/S. The accompanying dashed trend line shows the curve fitted to the FSC data points.

**Fig. 6:**
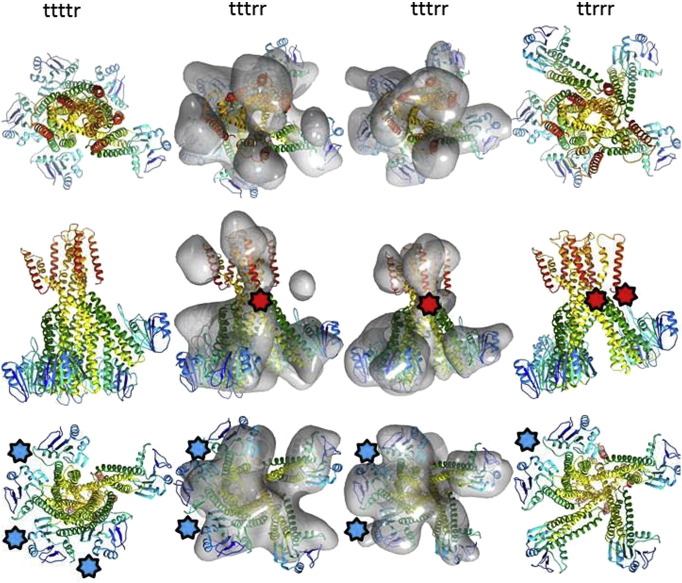
The final refined experimental MjCorA Cryo-EM map and the map refined from the docked composite model with ttttr, tttrr and ttrrr models of MjCorA: Cryo-EM maps are represented by grey semi-transparent surface, the contour level is set at the noise level of the map. The left-most column of panels shows orthogonal views of the ttttr model. The left-middle column shows the same views of the Cryo-EM map with the tttrr model docked. The right-middle map shows the map refined from the composite model again with the tttrr model docked. The right-most column shows three views of the ttrrr model (all protein representations as for the protomer in Fig. 1). The blue star indicates the close approach between adjacent cytoplasmic domains of t protomers. The red star indicates the close approach between the cytoplasmic domain of one r protomer and the adjacent TM domain of a second r protomer.

The most representative single starting envelope was therefore of the form tttrr. This envelope was refined with all of the data and an angular sampling interval of 6°. The lowest number of particles assigned to a class average in the final round of refinement was 11. The resolution was calculated as 25.4 Å (Fig. 5a). This map reveals an MjCorA structure which is considerably distorted from the crystal structure (Fig. 6). The conical cytoplasmic domain appears canted over and this is associated with a pronounced departure from C_5_ molecular symmetry.

### 3.5 Model fitting

The crystal structure of MjCorA (PDB ID: 4EV6) can be docked within the experimental map indicating a reasonable degree of fidelity of elements of the crystal structure with the map. However the partially dissociated subunits in the cytoplasmic domain and the separated lobes of density seen in the TM domain of the channel are clearly different from the crystal structure.

The approach taken in fitting coordinates within the map was sympathetic to the approach taken in refining the Experimental Cryo-EM maps described in Section 3.3. The crystal structure was docked into the map and the Cα coordinates of a single protomer were extracted. Two alternative orientations of this protomer were generated by tilting the protomer about the Cα coordinate of residue 250, corresponding to the narrowest part of the crystal structure. The axis for this tilt was the vector locating the pivot point from the C_5_ pore axis, thus minimizing steric collisions between tilted protomers. The two protomer tilts present in the two multirefined C_5_ symmetric maps described in Section 3.3 were estimated to be − 10 and + 20° relative to the conformation present in the crystal structure. These two rotations were applied to the protomer to represent the taut and relaxed state. Coordinates representing all channel protomers (chains A, B, C, D, E) were then generated for t and r states by subsequent rotations of α = 0.0, β = 0.0, γ = 0.0; 72.0; 144.0; 216.0; 288.0°, where α, β and γ are Eulerian rotation angles are defined with respect to the axes of the map.

One approach to refining from a model was to assign a fractional abundance to each t/r state such that the population states at each protomer location added to unity. A series of maps with different values of this relative abundance was calculated from the coordinate set above at a resolution comparable to the Cryo-EM map and a comparison made. Using this process the relative population values for the MjCorA protomers were estimated to be Chain:t/r = A:0.0/1.0, B:0.7/0.3, C:0.7/0.3, D:0.5/0.5, E:0.2/0.8. The numbers of degrees of freedom used in this method of assignment of the Cryo-EM density is limited: There are effectively 5 t and 5r protomers and 5 partition variables describing the relative abundance of t and r for each protomer. Refinement from this starting model gave an envelope similar to the experimental Cryo-EM map.

Each individual MjCorA pentamer must adopt one unique combination of (t/r) tilt angles. A consensus model may therefore be constructed by overlaying models corresponding to the allowed conformations seen in the exploratory Cryo-EM C_1_ asymmetric multi-refinements described in Section 3.3 (namely ttrrr, tttrr and ttttr, Fig. 4). These overlaid models are then weighted according to the proportion of particles assigned in a 3 way multi-refinement (namely 0.38:0.32:0.30). The number of degrees of freedom used in this method of assigning the Cyro-EM density is only slightly increased when compared to the earlier fractional abundance method; there are 9 t and 6 r protomers and 3 weights. The refinement of an envelope generated from these superimposed models against the particle data gave results similar to the experimental Cryo-EM map with marginally improved resolution (FSC_0.5_ = 24.9 Å, Supplementary Fig. 2) and more coherent density distributions (Fig. 6).

A refinement starting from a C_5_ symmetric version of the MjCorA crystal structure was undertaken using the same parameters. This produced an envelope similar in nature to both of the model-based refinements described above with a similar estimated resolution. However the resulting map was less clear. This lack of clarity can be explained as follows: The initial conformation of each protomer in the crystal structure lies between the t and r conformation. A refinement from this starting model leads to a final envelope representing an array of asymmetric MjCorA structures superimposed without pre-alignment. In contrast a refinement from three pre-superimposed asymmetric MjCorA (ttttr, tttrr and ttrrr) structures gives a final map where three of the five protomers remain closely superimposed (ttr) with the remaining two protomers adopting a weighted combination of t and r conformations.

In summary the experimental Cryo-EM envelope and those refined from the various models described show an asymmetric cytoplasmic domain and flaring of the TM domain. The most informative model was determined to be the combination of 3 asymmetric MjCorA pentamers of the form ttrrr, tttrr and ttttr.

## 4 Discussion

Crystals of wild type, full-length, CorA which diffract to sufficient resolution for detailed structural analysis have only been obtained for mutated protein or in high divalent ion concentrations. The analysis of a detergent solubilized CorA channel in solution allows much greater flexibility in the buffer conditions applied to the protein. The application of SAXS and Cryo-EM allows these solution conditions to be preserved during structural analysis. These techniques are hampered by low resolution, however with the aid of the higher resolution MjCorA crystal structure [12] new and functionally relevant information can be extracted. In the modelling procedure a distinction was made between more closely associated “t” protomers and less closely associated “r” protomers in a scheme reminiscent of the Monod, Wyman, Changeux (MWC) allosteric formalism [35]. Although no evidence of cooperativity in the binding of ions to the cytoplasmic domains of MjCorA can be derived from this study and the assumption of rotationally symmetric start and end structures implied in the MWC formalism is not present here.

The solution conditions have had a profound effect on the overall form of the MjCorA channel. The cytoplasmic domain appears to be canted over in a manner similar to the “bell” distortions observed in molecular dynamics simulations of TmCorA [17]. Based upon the conservative modelling procedure used to fit the Cryo-EM maps these distortions are not due to the concerted motion of the whole cytoplasmic domain but arise primarily from changes in the separation of adjacent domains (Fig. 6). An increase in cytoplasmic domain separation was also reported for the TmCorA molecular dynamics studies. The MjCorA structure reported here also shows that both increases and decreases in cytoplasmic domain separation occur and these conformational changes are communicated to the membrane spanning domains of the channel. The self-rotation function analysis of MjCorA crystal data is consistent with changes in domain orientations since correlation was observed over angles between 60 and 90°. A closer association of a subset of channel domains in the absence of Mg^2+^ may be a factor in the tetrameric quaternary structure observed for *E. coli* CorA extra-membranous domains [36], as in the study reported the domains were prepared in Mg^2+^ free buffer conditions.

The TM domain of MjCorA is encapsulated in DDM in this work. This detergent volume is not resolved in the final maps, as is the case with X-ray crystal structures and higher resolution Cryo-EM maps of membrane proteins [37]: This is presumably because detergent volumes are averaged away due to particle-to-particle variability of the overall form of the micelle. The individual TM helices are not resolved in the Cryo-EM map of MjCorA. The interaction of the TM domain with the micelle is qualitatively different from a lipid bi-layer, thus the spreading of the TM structure may be exaggerated in detergent solubilized MjCorA. However the envelope indicates that the asymmetry of the cytoplasmic domain is communicated to the TM structure and this may indicate a mechanical role of the TM domains in defining the admittance of the pore.

CorA is thought to operate as a channel [38] and therefore in order for the protein to be useful to the organism the free concentration of Mg^2+^ outside of the cytoplasm must be higher than that within the cell. Therefore *in vivo* the Mg^2+^ tension experienced by the TM domain is different to that of the cytoplasmic domain. Although this situation is impossible to replicate for a detergent solubilized channel, our study points to major magnesium-dependant changes in the channel structure which are of likely importance to the mechanism of channel gating. Each MjCorA protomer has an YGMNF motif in the first TM helix (Fig. 1, inset), these motifs are proposed to recognize Mg^2+^. The separation of the TM domains at low Mg^2+^ tension seen here indicates that Mg^2+^ stabilizes this TM oligomer, and that *in vivo* in the membrane the close association of TM domains seen in the crystal structures would be re-established by extracellular Mg^2+^.

The cytoplasmic domains of MjCorA are rich in both basic and acidic residues, both mono and divalent cations can associate with the acidic side chains of Aspartate and Glutamate. Of these possibilities polydentate interactions of Mg^2+^ with Asp, Glu and His residues are likely to have the greater affinity because of the summative effect of chelation leading to avidity. The concentration of free Na^+^ ions in the cell is in the mM range [39] and is therefore comparable to that of Mg^2+^. In both our studies and in other structural work on TmCorA and MjCorA Na^+^ ions are in excess. Aspartate and Glutamate side chains can also form salt bridges with Lysine and Arginine side chains. It is notable that the mutation to Alanine of two basic residues in the cytoplasmic domains of TmCorA appears to desensitize the channel to divalent cations yet neither the wild type nor mutated side chains could bind cations directly. These mutations do affect salt bridge formation [17]. This interpretation is also supported by the “allowed” structures in multi-refinement and in fitted models for MjCorA (Figs. 4c, 6). Taut conformations of an isolated protomer are not seen, taut conformations are present only with two or more adjacent protomers. This restriction suggests a direct interaction between adjacent protomers in the absence of Mg^2+^ which would be compatible with salt bridge formation.

The association of MjCorA channel cytoplasmic domains is modulated by the association of metal ions with Asp, Glu or His residues. These associations may be manipulated by changing the solution pH. In thermo-fluor assays it was demonstrated that the stability of the channel is affected by divalent metal ion concentration and pH [18]: In the assay the stability is inferred from the relative adsorption of a fluorescent probe by hydrophobic regions of MjCorA. It was found that for a given temperature a greater adsorption occurred both at lower pH (< 4.5), and in the absence of divalent cations. This observation is consistent with the increased hydrophobic surface afforded by the splayed TM domain structure observed in this study.

The solution conditions for both SAXS and Cryo-EM experiments were similar and in both cases with alkaline buffers lacking Mg^2+^ ions. Mg^2+^ and Co^2+^ ions were present in the crystallization buffers, but the pH of the crystallization buffer is calculated to be 4.3 suggesting that only 27% of carboxylate functions and less than 1% of imidazole side chains are deprotonated. This would lead to a low affinity of MjCorA for cations in the crystal. In addition the citrate buffer used in crystallization would compete with the protein for divalent cations [40]. It is therefore clear that the solution conditions employed in all of the studies reported here are unfavourable for the binding of Mg^2+^ ions to MjCorA. The information extracted from the crystallographic data was primarily used to confirm the quaternary structure of MjCorA (Fig. 2) and to estimate the structure factor obtained from SAXS at higher resolutions (Fig. 5) arising from structures on the intra-protomer scale, unaffected by the gross inter-protomer conformations modelled here.

This study indicates that when the free Mg^2+^ concentration is depleted, lower affinity interactions between the protomers of CorA, and between CorA residues and monovalent cations dominate. The protomer-protomer interactions are likely to include interactions between adjacent cytoplasmic domains, as well as interactions between the N-terminal domain and the C-terminal region of the TM domain of adjacent protomers noted in the work on TmCorA [17]. Our simple model(s) indicate that these interactions may be mutually exclusive: Looking down on the channel from the cytoplasmic side, cytoplasmic–cytoplasmic domain interactions are favoured with the clockwise protomer whereas N-terminal domain-TM domain interactions are favoured with the anticlockwise protomer (Fig. 6). These interactions would therefore be incompatible with the maintenance of C_5_ molecular symmetry and could drive conformational change in MjCorA.

CorA channels are proposed to recognize fully hydrated Mg^2+^ and admit partially dehydrated Mg^2+^ [38]. The most favourable interpretation of the ttttr, tttrr and ttrrr MjCorA structures assigned here would be that each represents one sub-conformation of the open channel. In the simple rigid body modelling applied here the r protomer conformation leads to an increased displacement of the YGMNF motif from the pore axis, whereas this displacement is reduced for t conformations. The limited resolution of this study prohibits any detailed conclusions about the exact dimensions of the pore of the channel. However an estimate of the relative admittance of the three states proposed in this study can be gained by comparing the mean displacement of YGMNF motif (as defined by the Cα of Glycine 278) from the pore axis. Normalized to the starting orientation for model fitting, as described in Section 3.5, these relative radii are ttttr = 0.7; tttrr 1.2; ttrrr = 1.6. Conformational change in the TM domain may also play a role in substituting the water of hydration of hydrated Mg^2+^ with coordinating species from the CorA protein. The separations between the TMVTT motifs indicated by our simple models are less varied compared to those of YGMNF, as the TMVTT motif is closer to the location where each protomer is pivoted in the modelling procedure. However the dispositions of the TMVTT motifs do change between the ttttr, tttrr and ttrrr and as such the motif may play a role in replicating the solvation shell of an Mg^2+^ ion within the TM domain. Thus changes in the coordination of Mg^2+^ ions by the TM domains are implied between the three models and this may play a pivotal role in ion conduction.

## 5 Conclusions

This Cryo-EM structure of MjCorA indicates that when depleted of Mg^2+^ the CorA structure adopts asymmetric conformation(s) in both the cytoplasmic and trans-membrane regions. The asymmetry of the cytoplasmic domain results from both increased and decreased separations of the channel protomers. The underlying drivers for change in channel conformation are likely to be due to two factors: (1) a replacement of chelating ions with monodentate ligating ions allowing increased protomer separation, and (2) the formation of salt bridges between protomers, acting to decrease protomer separation. Competing interactions between adjacent protomers induce the channel asymmetry.

The low resolution structure reported here is supportive of an emerging functional model of CorA channels where an asymmetric channel conformation results from the loss of divalent cations from the cytoplasmic domain of the channel. The study reported here indicates that this asymmetry would be consistent with an increased admittance by the TM segment of the MjCorA channel and improved access to ion binding sites between channel domains in the cytoplasm.

### Transparency Document

Transparency Document.
