# Supplementary material for: The Cryo-EM structure of the CorA channel from Methanocaldococcus jannaschii in low magnesium conditions
Source: Biochim Biophys Acta. 2015 Oct;1848(10Part A):2206–15. doi: 10.1016/j.bbamem.2015.06.002 (PMC4579555; doi:10.1016/j.bbamem.2015.06.002)
Supplement: Supplementary file 2 — Supplementary figures. [file mmc2.docx]

## Supplementary Material:


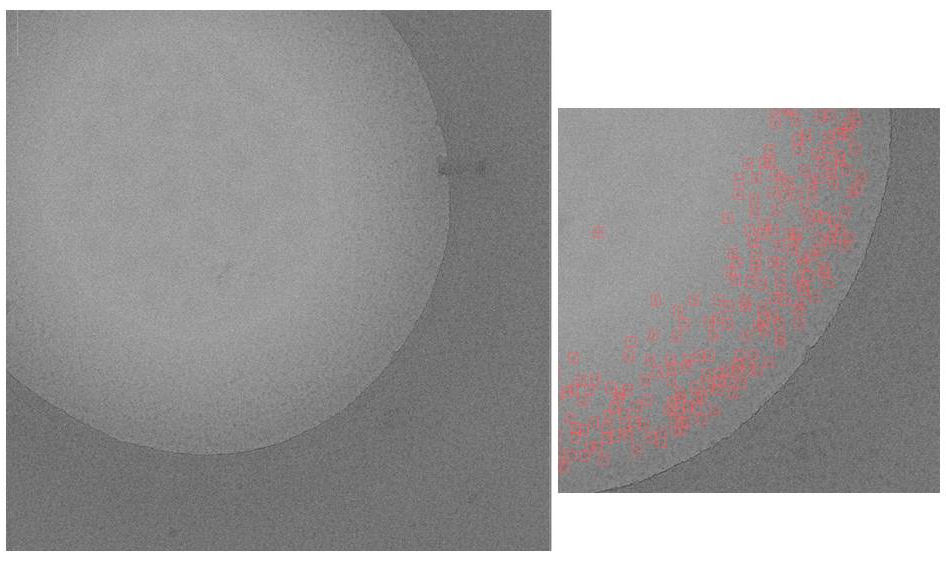


***Supplementary* Figure 1: A raw Cryo-EM micrograph shown alongside a magnified segment with automatically picked particles.** Boxes shown are 48 by 48 pixel^2^ corresponding to 168×168Å^2^.


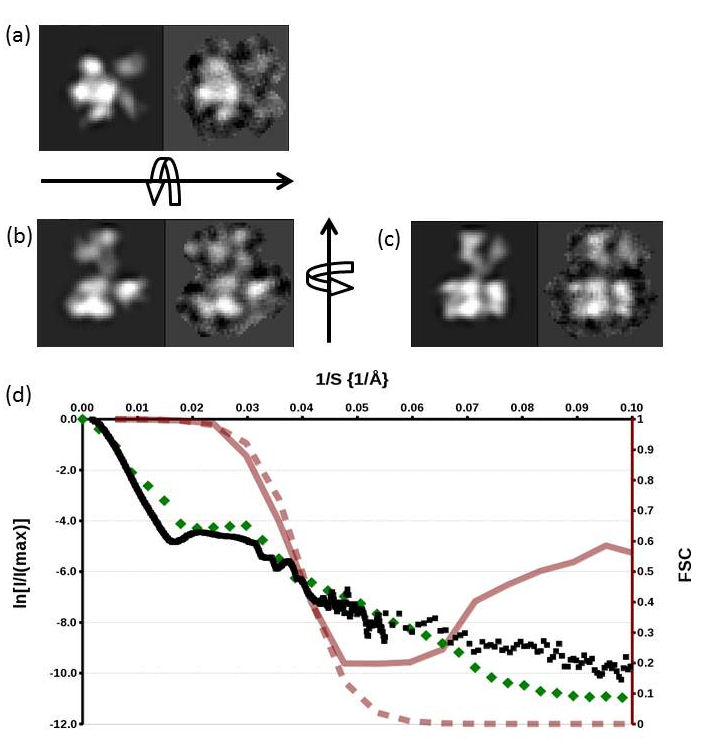


***Supplementary* Figure 2: The consistency of the Cryo-EM model refined from overlaid asymmetric (ttrrr, tttrr and ttttr) models.** (a-c) Paired back projections (left) and masked class averages (right) from the final round of refinement. Orthogonal views are shown with a view down the channel axis topmost. (d) A composite plot showing the agreement of the final model refined Cryo-EM map with the scattering and crystallographic data, and the resolution and of the map. SAXS data merged with spherically averaged crystallographic data are shown as black squares mapping to the left hand axis: These data are plotted as the natural logarithm of the normalized intensity. The sharpened structure factor from the final map (after application of a Debye-Waller sharpening “B” factor of magnitude 361) is plotted with green Diamonds. The semi-transparent solid, red line maps to the right hand ordinate axis connects the Fourier Shell Correlation (FSC) points of the unmasked final map plotted against 1/S. The dotted trend lines is the curve fitted to the FSC data points.
